# i-Rebound after Stroke-Eat for Health: Mediterranean Dietary Intervention Co-Design Using an Integrated Knowledge Translation Approach and the TIDieR Checklist

**DOI:** 10.3390/nu13041058

**Published:** 2021-03-24

**Authors:** Karly Zacharia, Amanda J. Patterson, Coralie English, Emily Ramage, Margaret Galloway, Meredith Burke, Raymond Gray, Lesley MacDonald-Wicks

**Affiliations:** 1 Faculty of Health & Medicine, School of Health Sciences, University of Newcastle, Callaghan, NSW 2305, Australia; amanda.patterson@newcastle.edu.au (A.J.P.); coralie.english@newcastle.edu.au (C.E.); emily.ramage@uon.edu.au (E.R.); margaret.galloway@newcastle.edu.au (M.G.); lesley.wicks@newcastle.edu.au (L.M.-W.); 2 Priority Research Centre for Stroke and Brain Injury, University of Newcastle, Callaghan, NSW 2305, Australia; 3 Hunter Medical Research Institute, Kookaburra Circuit, New Lambton Heights, NSW 2305, Australia; meredith@0412111111.com (M.B.); raymond.gray@newcastle.edu.au (R.G.); 4 Priority Research Centre for Physical Activity and Nutrition, University of Newcastle, Callaghan, NSW 2305, Australia; 5 Centre for Research Excellence in Stroke Rehabilitation and Brain Recovery, Florey Institute of Neuroscience and Mental Health, Melbourne, VIC 3052, Australia

**Keywords:** co-design, Mediterranean diet, telehealth, complex intervention, stroke, prevention, intervention development, IKT, TIDieR checklist

## Abstract

Lifestyle interventions to reduce second stroke risk are complex. For effective translation into practice, interventions must be specific to end-user needs and described in detail for replication. This study used an Integrated Knowledge Translation (IKT) approach and the Template for Intervention Description and Replication (TIDieR) checklist to co-design and describe a telehealth-delivered diet program for stroke survivors. Stroke survivors and carers (*n* = 6), specialist dietitians (*n* = 6) and an IKT research team (*n* = 8) participated in a 4-phase co-design process. Phase 1: the IKT team developed the research questions, and identified essential program elements and workshop strategies for effective co-design. Phase 2: Participant co-design workshops used persona and journey mapping to create user profiles to identify barriers and essential program elements. Phase 3: The IKT team mapped Phase 2 data to the TIDieR checklist and developed the intervention prototype. Phase 4: Co-design workshops were conducted to refine the prototype for trial. Rigorous IKT co-design fundamentally influenced intervention development. Modifications to the protocol based on participant input included ensuring that all resources were accessible to people with aphasia, an additional support framework and resources specific to outcome of stroke. The feasibility and safety of this intervention is currently being pilot tested (randomised controlled trial; 2019/ETH11533, ACTRN12620000189921).

## 1. Introduction

Knowledge translation is the process of moving research into clinical practice. A commonly cited research flaw is the 17 years that this process takes [1,2,3], that is if the research is implemented at all. It has been estimated that $85 billion of research funding each year is lost due to lack of translation into clinical practice [4,5]. The Stroke Recovery and Rehabilitation Roundtable (SRRR) was assembled to provide direction to move the field of rehabilitation research forward. The SRRR identified a lack of rigorous intervention development and detailed reporting as barriers to successful translation of research findings into practice [6]. To improve research impact, they endorsed the use of the Template for Intervention Description and Replication (TIDieR) checklist as a development and reporting guide to accurately report complex interventions, enhancing reproducibility and validity [7]. The SRRR also recommends using a co-design framework to ensure feasibility in a real-world context.

Both stroke and recurrent stroke are largely preventable because their risk factors can be modified by lifestyle changes. The INTERSTROKE study found that 10 potentially modifiable lifestyle factors were responsible for over 90% population attributable risk (PAR) [8]. Poor diet quality is one of those factors. However, the majority of the remaining nine factors (cardiac causes, waist-to-hip ratio, hypertension, psychosocial factors, diabetes, apolipoprotein ratio-ApoB/ApoA1) also have the potential to be addressed by improving diet quality.

Several dietary patterns have been shown to lower risk factors associated with stroke in clinical trials-Mediterranean diet [9,10], Nordic diet [11,12] and Dietary Approaches to Stop Hypertension (DASH) [13,14] diets. The only dietary pattern that has been shown to be effective in reducing actual risk (not just reduction in risk factors) is the Mediterranean diet. The PREDIMED study showed a Mediterranean diet supplemented with olive oil showed a 35% decrease in risk for primary stroke and the same diet supplemented with nuts showed a 45% decrease [15]. Due to its variety and adaptability, high adherence to a Mediterranean diet pattern has been consistently shown to be possible in countries outside of the Mediterranean [16,17,18,19].

We developed the AusMed diet program specifically for older Australians. The core food components of this are a high intake of fruit, vegetables, legumes, nuts and wholegrains; moderate intake of fish, poultry and dairy foods; limited intake of commercial sweets, red and processed meats; olive oil as the main source of culinary fats [20]. It was tested in a small population and the 2 weeks program was found to be acceptable to participants and resulted in high adherence (mean Mediterranean diet score [21] 9.6 ± 2.0 out of 14, where <5 is considered low, 6–8 moderate and ≥9 high adherence) [20]. It takes time for any change in diet to lower risk factors and improve health. Adherence needs to be long lasting to be meaningful and for that, interventions need to be created with the specific needs of their end users at the forefront.

Integrated Knowledge Translation (IKT) has at its core, a collaborative approach, where all who might benefit from an intervention (participants, researchers, health care providers and clinicians) are called knowledge users and are equally involved in its design from inception [22]. IKT has been shown to increase the applicability and impact of the research developed using its framework [23]. It has also been identified as a method to effect change in stroke care by bridging the gap between research and clinical practice [24]. A recent rapid review of co-design research found that while co-design is frequently used, there is a lack of standard terminology, the method is seldom described in detail and outcomes are not well defined [25].

Interventions developed to prevent recurrent stroke by changes in lifestyle are complex and have multiple components. Complex interventions are often not being described in enough detail to be of use in clinical practice [7]. Numerous public health frameworks have been proposed to address this need for better intervention development and reporting—for example, the Medical Research Council (MRC) [26] guidelines for complex interventions, the Consolidated Standards of Reporting Trials (CONSORT) Statement [27] and the TIDieR checklist [7]. Research tells us that few interventions are meeting the guidelines set out by these frameworks [28,29,30] and that has not improved over time [31]. In most cases, the research setting, recipient, provider and schedule of interventions are well described, but detailed descriptions of the actual content of the interventions and supporting materials or equipment used are often lacking [28,29,30,32]. Frequently missing were adequate reporting of intervention development, content and materials and treatment fidelity [31].

The IKT co-design framework has been used to develop a supervised physical activity intervention delivered via telehealth for stroke survivors (i-Rebound after Stroke—Let’s get Moving) [33]. The dietary intervention developed by this study (i-Rebound after Stroke—Eat for Health) is being piloted alongside this as part of the ENAbLE pilot trial to reduce secondary stroke (2019/ETH11533 and ACTRN12620000189921). The aim of this study was to develop a Mediterranean dietary intervention (using the AusMed diet as a framework) for the prevention of second stroke using the TIDieR checklist for reporting (including the National Institute of Health Treatment Fidelity Framework [34]) and IKT co-design to improve its replicability and feasibility.

## 2. Materials and Methods

**Figure 1:**
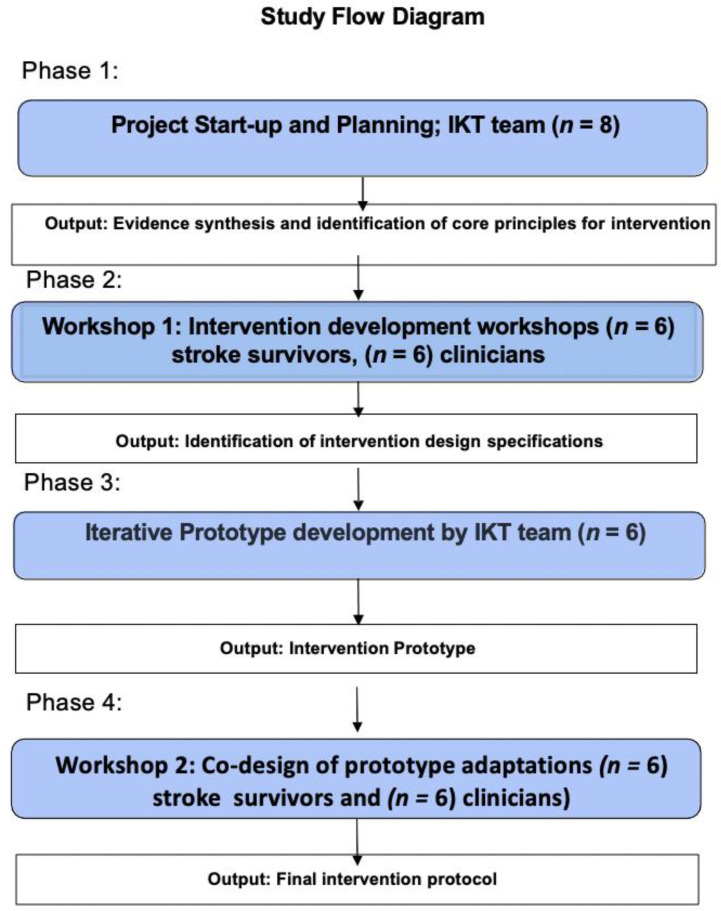
Flow chart of Integrated Knowledge Translation (IKT) study processes.

This study used the IKT methodology (conducted over 4 phases). The study process is described by Figure 1. Workshops were conducted to codesign a Mediterranean dietary intervention to be delivered via telehealth for secondary stroke risk reduction. This approach complies with both the SRRR roundtable recommendations and the TIDieR framework for complex intervention reporting, incorporating the NIH Treatment Fidelity Framework at all stages of development.

### 2.1. Recruitment

The IKT research team (*n* = 8) was already in place. Purposive recruitment was utilised to ensure a breadth of experience across knowledge users. Participants were identified as possible end users of the program (stroke survivors, carers and dietitians responsible for program delivery) and were recruited from a variety of sources; from the initial ENAbLE trial codesign process (*n* = 4), utilizing professional networks (*n* = 6) and through word of mouth (*n* = 2). Written and informed consent was obtained from all participants.

### 2.2. Phase 1: Project Start up and Planning

The IKT research team consisted of researchers (*n* = 3), clinicians with experience in stroke rehabilitation and/or telehealth delivery (*n* = 3) and stroke survivors (*n* = 2). As the physical activity intervention to be trialled alongside this dietary intervention was already in place, the timeframe from Phase 1 to finalised intervention prototype was identified as 8 weeks. An iterative collaborative process was used to identify the essential elements of the program and define the research questions. Strategies to engage participants in the co-design process were identified to capture a breadth of information applicable to a variety of stroke outcomes.

### 2.3. Phase 2: Workshop 1. Intervention Development

A series of stakeholder workshops were held with stroke survivors (*n* = 4), carers (*n* = 2) and specialist disability dietitians (*n* = 6). Stroke survivor and clinician workshops were held separately. Researchers used persona [35] and journey mapping [36] processes to identify essential items to the intervention design. Persona and journey mapping are tools used frequently in marketing to explore a user’s experience of a program or product in order to improve it, similar to a case study [37]. Two personas of stroke survivors were developed by the research team using data derived from the Australian Institute of Health and Welfare (AIHW) [38] to represent common stroke-related impairments that may affect the ability to adhere to a diet program (Table S1). Persona characteristics were intentionally varied (different genders, employment status, support structures and stroke outcomes). The facilitator used a semi-structured question guide to describe the AusMed diet program in its original format, which included a resource booklet with Mediterranean diet education, recipes and a checklist, a 2 h group information session and a 2 weeks example meal plan and shopping list. Workshop facilitators mapped the journey of each persona through the program. Participants were asked to identify how the persona might complete the program, to describe enablers and barriers for each persona and identify essential elements needed for effective and feasible diet program adherence. Each workshop was audio recorded, transcribed and thematically analysed. Analysis was emailed to participants for member checking which gives them the opportunity to review and clarify any information or give additional data.

### 2.4. Phase 3: Prototype Development Using TIDieR

Essential elements and detailed data collected from the workshops were mapped to the 12 items of the TIDieR checklist (Table 1) and used to develop the intervention manual, resources and components necessary for the intervention prototype. The research team then used the NIH Treatment Fidelity Framework to develop a fidelity checklist to ensure the intervention will be delivered as intended.

### 2.5. Phase 4: Co-Design of Prototype Adaptations

Another series of workshops was then held with knowledge users (*n* = 6 stroke survivors and carers and *n* = 6 dietitians) to refine and adapt the intervention prototype. Prior to the workshop, the prototype and resources were emailed to participants to allow time for formulation of feedback. The workshop facilitator summarised the thematic analysis from workshop 1 and presented the outcome developed as a result. A physical copy of the emailed prototype resources was also presented to participants. Participants feedback was sought after each section of the presentation. Each workshop was audio recorded, transcribed and thematically analysed by a member of the research team as per the six-phase guide outlined by Braun and Clarke [39]. Analysis was then emailed to participants for member checking. The data collected were then used to finalise the TIDieR checklist and intervention materials ready for trial.

## 3. Results

### 3.1. Phase 1: Project Start up and Planning

Iterative collaboration meetings, a literature search and prior research by the IKT team identified essential elements to be included in the intervention protocol, the research questions to be answered by the Phase 2 co-design workshops and strategies to facilitate co-design.

The key elements identified were the use of the Mediterranean diet pattern (modelled on the AusMed framework), individual dietary counselling sessions, delivery by telehealth using individual video calls and embedding behaviour change techniques within each session using the Behaviour Change Wheel [40].

To capture a broad scope of data, two personas were developed to represent differing stroke outcomes, gender, support needs and demographics. The research questions to be answered by the codesign process are displayed in the table below (Table 1).

### 3.2. Phase 2: Workshop 1 Intervention Development

The IKT research team identified the co-design of the program elements and structure as the goal of the workshops with the data collected to be mapped to the TIDieR checklist to ensure complete intervention development. The use of journey mapping gave the workshops focus and direction and enhanced participant engagement with the codesign process. The variety of characteristics of the developed persona meant that barriers and enablers described were more broadly applicable than would otherwise have been captured by the experience of workshop participants.

Thematic analysis of workshop transcripts (Table 2) identified five elements that stroke survivors and dietitians found were key to the successful delivery of the AusMed diet program: inclusion of a detailed initial assessment, accessible resource design, development of resources specific to stroke outcome (such as one-handed food preparation, fatigue strategies, convenience meals), creation of a support framework (including optional group and peer support, text messages and reminders) and resources to support effective telehealth delivery.

### 3.3. Phase 3: Intervention Prototype Development

The IKT team combined initial essential elements identified in Phase 1 with data from the Phase 2 co-design workshops and mapped this to the TIDieR checklist, thereby developing the intervention protocol and supporting resources. A complete description of the intervention developed by the IKT co-design process is outlined in the TIDieR checklist (Table 3).

As a result of the co-design process, there are six essential elements to applying the i-Rebound after Stroke-Eat for Health program;

1. The end goal of the program is for participants to achieve high adherence to a Mediterranean dietary pattern.
2. Delivery is via individual/tailored diet counselling sessions.
3. Sessions are to be delivered by video call to the participant’s own home.
4. Each session must include embedded behaviour change strategies to support participants’ long-term adherence.
5. All resources must be accessible for all (including in a language and a format that are aphasia friendly) and be able to be tailored to different stroke-related impairments.
6. The intervention must include optional, embedded support systems such as peer support groups and/or text messages to facilitate behaviour change.

The intervention protocol, which will be delivered by an Accredited Practicing Dietitian (APD), consists of 10 individual diet counselling sessions to be delivered over 6 months. The first two sessions (weekly) focus on detailed initial assessment, program education, current diet history and assessment of barriers to change. The next five sessions are fortnightly and aim to achieve high adherence to a Mediterranean diet pattern. All sessions have the same format; revision of previous goals, Mediterranean diet score calculation, identification of new goals, assessment of barriers, identification of behaviour change strategies to enable and resources required to support. The final three sessions are delivered monthly and aim for self-efficacy in maintaining dietary change.

Supporting resources were developed as a result of the co-design process utilising aphasia-friendly design guidelines with appropriate font size and type, spacing and visual representation; pictures and icons [53]. A participant program manual and resources were developed to support a variety of stroke outcomes and address barriers to change; fatigue, convenience meals, one-handed food preparation, templates for meal plans, shopping lists, goals setting. Also developed was a recipe book and a practitioner training manual including resources for effective communication via video call, technology instructions, behaviour change taxonomy to be used in session and a telehealth session manual to be used by the therapist for each participant—this includes session outlines, a validated 14-point Mediterranean diet score [54] to assess adherence and fidelity checklist (Table 4). The therapist and telehealth manuals will be used to train clinicians in delivery of the program to ensure fidelity.

Co-design identified the need for resources to be specific to the needs of the individual. Additional resources will be developed according to need as identified within individual sessions. This will result in a bank of resources being compiled over the duration of the pilot trial for use in a larger roll out. All support sessions and text messaging are optional and will be tailored to individual need or request.

As part of the TIDieR framework, a fidelity assessment plan was produced using the National Institute of Health’s Treatment Fidelity Framework [34]. Fidelity is measured by the five domains of this framework; 1. design, 2. training, 3. delivery, 4. receipt and 5. enactment. (See Table 2 for a detailed description). Data will be collected and managed using Research Electronic Data Capture (REDCap) [56,57] hosted at the Hunter Medical Research Institute (HMRI).

### 3.4. Phase 4: Co-Design of Intervention Prototype Adaptations

The intervention prototype was presented for participant feedback at Phase 4 co-design workshops. All workshop participants agreed that the intervention prototype developed as a result of the co-design workshops was acceptable and feasible.

“Yeah. It’s easy to read, easy to understand. Excellent. Yeah. You’ve really got everything in there.” E.

“You’ve got some lovely, very simple recipes. Excellent resources applicable for a variety of clients. That actually looks deliverable…The best I’ve seen for a while. You’ve got me. It’s a really, really good book.” J.

Thematic analysis of transcribed interviews revealed three overarching themes to refine the intervention. The first was development of carer-specific resources; several clinicians suggested resources created to allow carers to facilitate opportunities for independence would be beneficial.

“You can often be more prescriptive with carers. Outline specific duties that you want them to undertake that will facilitate the behaviour change for the client. Things like kitchen preparation set up or meal preparation where the client will finish the meal off. Or teaching them to facilitate the meal planning and shopping with the client.” L.

The second was demonstration; stroke survivors re-iterated the importance of seeing themselves or someone like them demonstrate preparation skills. Video was seen as an important tool for self-efficacy.

“I think that at the very least, having videos by us that teach people how to do those things is great. If you made little demonstration videos, like of various things you need to do like prep set up or, or even for one recipe and now you’ve got to chop, you’ve got to blend, you got to stir it. This is how you can make it easier.” M.

The final theme identified was for support sessions to be themed and guided to allow time for participants to prepare their input for peer support. All participants emphasised that peer support and sharing their experience was an important method of skill building for them.

“Make them as useful as you can. You know, you’re your own best resource and your each other’s own best resource as well. Maybe like, you choose a theme for each session and then bring your best ideas for the group for that session. So, the theme of this week’s group session is going to be, how you open packets and cans.” R.

The final prototype was then revised by the IKT team to reflect participant adaptations with some ideas parked for future use due to time constraints. The intervention is currently in the pilot trial phase, with ongoing refinement.

## 4. Discussion

Lifestyle interventions to lower second stroke risk are inherently complex. Understanding their development process is essential for replication and translation into clinical practice yet rarely is this reported with enough detail to be effective [28,29,30,31]. The aim of this study was to use the IKT framework and TIDieR checklist with the NIH Treatment fidelity Framework to co-design and describe a Mediterranean dietary intervention specifically for stroke survivors. While the Stroke Recovery and Rehabilitation Roundtable (SRRR) recommends the use of the TIDieR checklist in reporting interventions [6], this study also used it as a framework to guide development. The checklist gave clear justification for and reliable description of all intervention components and ensured complete intervention development from the outset. Using both IKT and the TIDieR checklist allowed for a complete description of the diet program methodology using standardised terminology addressing a cited limitation of co-design research [25].

Both the SRRR and a recent Cochrane review identify co-design as necessary for intervention acceptability and feasibility both in trial and clinical practice [6,58]. Enablers to successful co-design using IKT include having multiple opportunities for interaction, a phased approach for iterative development, formalised processes and the establishment of partnership early in the process [59]. This intervention development timeframe was brief (8 weeks) in comparison to the same IKT process undertaken by the research team to develop the physical activity intervention (i-Rebound after Stroke-Let’s Get Moving) which took 7 months. However, a number of stakeholders participated across both studies [33]. As a result, relationships were already formed, and phased study processes formalised. Strategies were put in place to streamline development; the AusMed diet program was used as a framework for redevelopment rather than to design a completely new intervention, separate workshops were held for clinicians and stroke survivors, and persona and journey mapping used to guide those workshops. These strategies improved workshop focus and augmented the development of an intervention acceptable to both clinicians and stroke survivors within the required timeframe.

The IKT process was used to finalise an intervention prototype with six key elements. While not all of these elements are unique to the stroke survivor population, collectively, they address barriers to dietary change and intervention delivery that are unique to stroke survivors. Stroke causes a greater range of disabilities than any other condition. Debilitating fatigue affects up to 75% of stroke survivors [60,61], cognitive difficulty such as aphasia affects 40–50% [62,63] and physical disability up to 65% [64,65]. These outcomes make access and participation in standard diet programs to lower the risk of second stroke difficult [66]. Co-design highlighted key program elements for inclusion that address these barriers; delivery by telehealth to improve access, tailoring of the intervention by individual diet counselling and resource creation, embedding behaviour change techniques using the Behaviour Change Wheel [52] and support within the intervention processes and resources. A recent Cochrane review on self-management programs for quality of life in people with stroke found that in order to create effective programs that are meaningful and acceptable to people with stroke, stroke survivors need to be involved in their design, the interventions needs to be tailored to their ability and delivered by professionals and peers that are experts in stroke and its consequences [58].

Unique to this study is the development of accessible intervention components and resources designed specifically to address the barriers created by stroke outcome. Aphasia can significantly alter the understanding of language, speech and writing ability [62,63,67]. Lifestyle intervention programs often rely on written resources to enable participant adherence. In order for these to be useful to someone with aphasia, specific design guidelines must be used to ensure readability [53]. Materials must be designed using easily readable sans serif fonts (minimum 12–14 pts), 1.5–2 spacing, use of images and lots of white space on the page. Program resource materials were created to support participants with a variety of stroke outcomes (such as strategies for fatigue, adaptations for cooking with muscle weakness or paralysis, flavour without salt). All were developed using these design principles to improve accessibility and acceptability.

The SRRR recommends adoption of a fidelity framework in the intervention design phase to help identify which aspects are most effective and which need refining [6]. Using the National Institute of Health Fidelity Framework to design a fidelity plan has given this intervention a detailed description of delivery and audit processes to ensure it can be done well in any setting by any qualified therapist. The fidelity plan includes not only session data and behaviour change coding but also recruitment rate and retention. Treatment fidelity ensures that any outcome is a direct result of the intervention which adds to scientific confidence.

Interventions with high treatment fidelity have also been shown to have improved treatment outcomes [68]. Embedding measurement of fidelity from the beginning will improve study processes and translates into a rigorously designed trial.

## 5. Limitations

Several limitations must be noted when interpreting the study process described here. As stated, the timeframe between intervention development and finalisation for pilot trial was 8 weeks. This study shows that it is possible to co-design an intervention using the IKT process within that short time frame. However, time constraints limited the number of stakeholders involved and resulted in more structured workshop sessions, which may have resulted in fewer ideas from outside of that structure. Further workshops involving more participants, across a wider range of allied health disciplines (such as occupational and speech therapy) and representative of a variety of stroke outcomes, would have been of benefit to confirm the key themes and essential elements of the intervention. The facilitator of the workshops was also responsible for data analysis, which was valuable in terms of intervention development, as they had participated in discussions, but may provide a source of bias. Including independent data analysis would help prevent this.

## 6. Conclusions

Promoting lifestyle interventions aimed at lowering risk factors for stroke seems intuitively appropriate yet few stroke survivors are adhering to them [69]. A rigorously developed intervention, specific to the needs of both the people who will benefit from AND the people who will deliver the intervention will make long-term adherence more likely in a real-world setting. IKT uses the real-world experience and contexts of knowledge users to ensure that intervention design is both effective and achievable in practice. Creating interventions that are acceptable, feasible and replicable could improve translation and decrease money lost from underuse of interventions [4,5].

A recent Cochrane review looking at primary and secondary prevention of cardiovascular disease by the Mediterranean dietary pattern found that while observational studies confirmed the benefits, there was a distinct lack of trial evidence [70]. The intervention developed by this study is currently in pilot trial, testing feasibility and safety, with a process evaluation being conducted concurrently, which will add to trial evidence. The key elements developed by the co-design process and the detailed intervention description will allow for improved feasibility, sustainability and translation.

The focus of this article was to describe the development process of a novel, complex, Mediterranean dietary intervention for stroke survivors. The four phases of the IKT framework identified essential elements for inclusion and enabled a comprehensive description of the intervention using the TIDieR checklist. This process will provide a template to help researchers replicate the intervention with a view to closing the gap moving stroke research evidence into clinical practice.

## Figures and Tables

**Table 1:** Research Questions Resulting from Phase 1.

Phase 1 Research Questions
1. What are the stakeholders perceived barriers to a Mediterranean diet program delivered via telehealth?
2. What are the stakeholders perceived facilitators to a Mediterranean diet program delivered via telehealth?
3. What do the stakeholders perceive as essential elements to include in a Mediterranean diet program to be delivered via telehealth?

**Table 2:** Thematic Analysis of Workshop 1 (*n* = 12).

	Essential Criteria	Verbatim Evidence
Initial assessment	Current barriers Physical ability Environment Support accessed Changes made prior to assessment Appointment burden	“Everybody has a different stroke, everybody has a different life experience, everybody has a different disability. We’re all one of a kind.” M. “things to consider are kitchen layout, oven height, tap turning…1/2 or ¾, fridge placement, capacity, storage, you need to know what you’re working with.” L.
Resource Design	Aphasia friendly; large (14 pt) San serif font, 1.5–2 spacing, minimal text, bold Visual—photographs and icons Paper + video format People pictured should be stroke survivors Graded levels of support	“If it’s too hard to look at then it’s just too tiring. I won’t use it.” R. “Videos that show us showing people how to chop things or how to peel a carrot. Show me how to do it.” R.
Supporting resources	Dietary education: stroke specific Fatigue strategies Hemiplegia-specific food preparation support Flavour without salt Staple/convenience foods ideas Templates: self-efficacy	“Pantry staples and convenience meals where you’re compiling rather than preparing.” L. “I don’t want you to do it for me, I want you to teach me how to do it for myself.”R. “I struggle with taste.” S.
Support framework	Morning sessions: due to fatigue Session reminders and text message support Peer support: closed Facebook group, group telehealth, virtual suggestion box All support to be optional	“Honestly, the best part is the cup of tea after the session. You learn more from other people’s experience of their stroke and how they do things.” F. “It all depends on the person and what they want and need. You’d have to put your hand up for it.” B.
Telehealth requirements	Clinician manual Technology and environment set up Aphasia-specific advice: eye contact, time for thought and reply Screensharing for tailoring resources	“Having a double monitor set up and screensharing so you can tailor resources in real time.” M. “I need time to think about things.” R.

Participants included *n* = 4 stroke survivors, *n* = 2 carers, and *n* = 6 dietitians.

**Table 3:** TIDieR Checklist.

TIDieR Item	Description
**1. Brief Name** Provide the name or a phrase that describes the intervention	i-Rebound after Stroke—Eat for Health
**2. Why** Describe the rationale, theory or goal of the elements essential to the intervention	This intervention contains 6 essential elements. Mediterranean diet pattern has been shown to lower the risk of stroke by between 35 and 45% [15] and due to its variety and adaptability, high adherence has consistently been shown outside of the Mediterranean [17,18,19,20,41]. Individual diet counselling to tailor intervention delivery to the participant’s circumstance and level of ability post-stroke. Tailoring advice has been shown to improve diet quality and lower chronic disease risk [42,43,44]. Telehealth/video call to be used to deliver diet counselling session by an Accredited Practicing Dietitian. Equitable access to suitable prevention programs is a barrier to lowering stroke risk [45,46]. Telehealth is an effective and economically viable alternative to a face-to-face model of care [47,48] and has been shown to improve diet quality and clinical outcomes associated with stroke risk [49]. Embedded behaviour change strategies are essential to support dietary change [50,51]. Michie’s Behaviour Change Wheel (BCW) addresses capability, opportunity and motivation to change behaviour [52] and central to the intervention is the use of this taxonomy within counselling sessions, in resources and within embedded support. Accessible resources; stroke outcome can make it difficult to adhere to dietary change. Outcomes such as hemiplegia, aphasia and fatigue can make food provision and preparation challenging. To support dietary change. Creating resources with design specification to address barriers. Embedded support both from program administration (text messages with embedded BCTs) and peers (program administered participant Facebook group) to support behaviour change will ensure adherence.
**3. What: Materials** Describe any physical or informational materials used in the intervention, including those provided to participants or used in intervention delivery or in training of intervention providers. Provide information on where the materials can be accessed	Participant resource manual Aphasia-friendly design with appropriate fonts, size and spacing, use of white space and images. Mediterranean diet education. Program outline. Telehealth access instructions. Support resources; convenience meals, fatigue tips, pantry staples, flavour without salt, goal setting templates, meal plan examples and template, food preparation tips for one-handed cooks. Participant recipe book Aphasia-friendly design. 40 recipes designed to complement Australian seasonal produce and foods familiar to population. Recipes are colour coded to match meal type (e.g., breakfast is blue and lunch is green). Practitioner Training manual Program outline and rationale. Telehealth resource for effective diet counselling via telehealth. Behaviour change taxonomy and strategies to support participant behaviour change. Telehealth session manual Session checklist for fidelity. Initial session assessment template; collecting data on stroke experience and current diet. Mediterranean diet score template. Session notes template. Contact first author for further details and access to program materials.
**4. What: Procedures** Describe each of the procedures, activities and/or processes used in the intervention, including any enabling or support activities	Individual diet counselling 10× Individual diet counselling sessions delivered via telehealth video call. The goal of these sessions is to achieve adherence to a Mediterranean diet pattern. First 3 months goal: to attain high adherence (score of 9 or above on validated 14-pt Mediterranean diet score [21]); final three months: self-efficacy, confidence to continue long term. Initial session—detailed participant information collection of stroke history, co-morbidities, current diet assessed against Mediterranean diet score, assessment of barriers. Second session—stroke diet–disease relationship, Mediterranean diet pattern education, Mediterranean diet score, goal identification, assessment of barriers, identification of strategies to change behaviour, identify resources required to support change. Subsequent sessions—assessment of previous weeks’ goals, Mediterranean diet score, identification of new goals, strategies to achieve goals using Behaviour Change Wheel taxonomy. After each session, the participant is emailed a session summary with any resources attached. Each participant is emailed a reminder prior to the next session. Text message support Optional text messages delivered at a frequency requested by the participant, using Behaviour Change Wheel taxonomy to support goals. Group support Optional private Facebook group moderated by study dietitian for peer support.
**5. Who Provided** For each category of intervention provider (e.g., psychologist, nursing assistant), describe their expertise, background and any specific training given	Intervention in this pilot study will be provided by a single Accredited Practicing Dietitian (APD) (first author, 2 years’ experience in cardiovascular disease prevention and disability). For future application, the detailed practitioner training manual and telehealth session manual will ensure the fidelity of treatment and form the basis for other APDs to provide the intervention.
**6. How** Describe the modes of delivery (e.g., face to face or by some other mechanism, such as the internet or telephone) of the intervention and whether it will be provided individually or in a group	Individual sessions delivered via telehealth (video call), text messaging to support goals; optional and delivered at a frequency negotiated with participant, Facebook group support; optional, usage dependent upon participant engagement with the platform.
**7. Where** Describe the type of location where the intervention occurred, including any necessary infrastructure or relevant features	The intervention will be delivered using video calls to the participant’s own home. Participant requirements Internet connection and device (desktop, laptop, iPad or phone) with webcam and microphone capability. Therapist requirements Internet connection and device with webcam and microphone capability (preferably two monitors to allow for screen sharing of resources), headphones.
**8. When and How Much** Describe the number of times the intervention was delivered and over what period of time including the number of sessions, their schedule, duration, intensity or dose	The intervention will be delivered over a 6 month period. There will be 10 individual, 1 h diet counselling sessions. Weeks 1–2 will be delivered weekly, with the remaining 5 sessions of the first 3 months to be delivered fortnightly. The last 3 months of the intervention will be delivered monthly.
**9. Tailoring** If the intervention was planned to be personalised, titrated or adapted, then describe what, why, when and how	Goals and strategies tailored to participant’s ability and choice of goal. All resources are created in pdf templates to allow for individual tailoring. Intervention delivery is to be titrated, and the focus of the first 3 months is on increasing adherence to Mediterranean dietary pattern and improvement of overall diet quality. The focus of the final 3 months is on strategies for self-efficacy to ensure long-term behaviour change.
**10. Modifications** If the intervention was modified during the course of the study, describe the changes (what, why, when and how)	N/A Intervention is currently in pilot stage. Any modifications to protocol data are being collected and will be reported in process evaluation.
**11. How Well: Planned** If intervention adherence or fidelity was assessed, describe how and by whom, and if any strategies were used to maintain or improve fidelity, describe them	A fidelity plan has been designed and will be assessed according to the 5 domains of the National Institute of Health (NIH) Treatment Fidelity Framework. The detailed fidelity plan is described in Table 3.
**12. How Well: Actual** If intervention adherence or fidelity was assessed describe the extent to which the intervention was delivered as planned	N/A Intervention is currently in pilot stage. Fidelity will be reported as part of feasibility study.

**Table 4:** NIH Treatment Fidelity Plan.

Domain	Measure
Design	Measured by adherence to the TIDieR checklist
Training	The pilot will be conducted by KZ; a training manual has been developed for use post-pilot. The manual includes Standard session protocol, guided by BCW taxonomy of behaviour change [40]; Telehealth resources—session guide, technology instructions; Standardised training will include Healthy Conversation Skills [55] training, 1 h training workshop, mock telehealth consultation, supervision and support.
Delivery	A Redcap database has been developed to capture delivery data. Recruitment data; Session attendance and duration; Adherence to session protocol; Five-point Likert scale to assess dietitian’s perception of participant, understanding of content, interest/attention in session, active participation in goal setting/strategies; Adverse events; Session audit: selection of telehealth sessions to be recorded and mapped to the BCW taxonomy.
Receipt/enactment	Process evaluation satisfaction survey, Qualitative individual interviews for detailed feedback and participant satisfaction.

## Data Availability

The data presented in this study are available upon reasonable request to the corresponding author. The data are not publicly available due to confidentiality reasons.

## Supplementary Materials

The following are available online at https://www.mdpi.com/article/10.3390/nu13041058/s1, Table S1: Description of Persona used in Workshop 1.
