# Supplementary material for: i-Rebound after Stroke-Eat for Health: Mediterranean Dietary Intervention Co-Design Using an Integrated Knowledge Translation Approach and the TIDieR Checklist
Source: Nutrients. 2021 Mar 24;13(4):1058. doi: 10.3390/nu13041058 (PMC8064089; doi:10.3390/nu13041058)
Supplement: Supplementary file 1 [file nutrients-13-01058-s001.pdf]

Supplementary Material

Table S1. Description of Persona used in Workshop 1.

Persona 1.

**Demographic information**	• **Male**<br>• **45**<br>• **Lives at home with wife and 2 children (12 & 14)**
**Employment**	**Associate Professor: Biomedical Science**<br>**Returned to work part time - 2017**
**Stroke History**	**2015 - Carotid Artery Tear followed by stroke**
**Stroke outcome**	• **Broca's aphasia**<br>• **Finds speech difficult**<br>• **Understands words being spoken**<br>• **Finds writing more difficult to read**<br>• **Tires easily**

Persona 2.

**Demographic information**	• **Female**<br>• **65**<br>• **Lives alone**<br>• **Has family support (daughter lives near-by)**<br>• **Has carer support through National Disability Insurance Scheme (NDIS) – 1 day each week**
**Employment**	**Retired**
**Stroke History**	**2008-2010 - several TIA's followed by major stroke in 2010**
**Stroke outcome**	• **Partial right-sided hemiplegia**<br>• **Walks with aid**<br>• **Speech and swallowing recovered with rehabilitation**<br>• **Decreased hand-grip function**<br>• **Arm feels heavy, wrists are weak**<br>• **Tires easily**
